# Understanding Pilots' Perceptions of Mental Health Issues: A Qualitative Phenomenological Investigation Among Airline Pilots in the United States

**DOI:** 10.7759/cureus.66277

**Published:** 2024-08-06

**Authors:** David S Cross, Ryan Wallace, James Cross, Flavio Coimbra Mendonca

**Affiliations:** 1 Aviation, Embry-Riddle Aeronautical University, Daytona Beach, USA; 2 Educational Affairs, Drexel University College of Medicine, Philadelphia, USA

**Keywords:** reporting, stigma, mental health, aviation, pilot

## Abstract

Background and objective

Although mental health is always a major concern, particularly for airline pilots, knowledge of and attitudes toward mental health have not always been emphasized for safe operations in the aviation industry. Fear of self-reporting, stigmas, and lack of knowledge about mental health conditions are prevalent in this industry. The purpose of our research was to examine pilots' perceptions of mental health issues, the resources available to them, and the reasons they may or may not report these issues.

Methods

We conducted a qualitative, phenomenological study in which 21 commercial pilots were interviewed to better understand their perceptions of mental health issues, available self-help resources, and rationale for failing to report mental health issues.

Results

The results of our analysis using NVivo software showed that pilots neither reported the issues nor trusted the processes meant to address mental health issues. Three themes emerged from the research: (1) pilots avoid discussing mental health issues for fear of repercussions, (2) although resources exist, pilots generally distrust the confidentiality of reporting systems, and (3) pilots honestly believe that reporting any mental health issue will be devastating to their careers.

Conclusions

Airline companies and the Federal Aviation Administration (FAA) need to change processes and instill a sense of trust in reporting systems among pilots so that they feel safe reporting mental health concerns and receive improved treatment. This can lead to more accurate reporting of conditions and ensure safe flight operations.

## Introduction

On March 24, 2015, Germanwings Flight 4U9525 crashed in the French Alps [1]. The French Bureau of Enquiry and Analysis for Civil Aviation Safety concluded that “the aircraft was intentionally brought down by the co-pilot” [1]. More than 5,000 pilots are suspected of hiding major health issues, and most of them are still flying [2]. Conditions including anxiety, depression, fatigue, and stress are all connected with poor mental health in aviation [3]. The unique stressors of the aviation industry, including excessive work hours, irregular schedules, and the responsibility for passenger safety, contribute to mental health challenges among aviation professionals. Understanding and mitigating these challenges is crucial for fostering a work environment that supports the mental well-being of individuals in key aviation roles [4].

The analysis of literature sources addressing the issue of mental health among aviation employees shows that pilots pay little attention to their own mental health or that of other pilots [3]. However, their health is pivotal as it affects their effectiveness and ability to provide reliable, safe services to customers. Silva et al. analyzed a survey of 182 pilots and found that more than 10% of pilots experience depression [3]. Most aviation accidents occur due to human errors that may be influenced by mental health factors, including stress or fatigue [3]. Hence, human error usually refers to an accident where an individual’s action or decision was causal or a key contributing factor [5]. Mental health and well-being are critical constituents of the safe performance of pilots and other aviation employees. According to the Federal Aviation Administration (FAA), many pilots who suffer from mental health issues in the US must make the difficult choice of whether to disclose their diagnosis, thereby risking the loss of their medical certificate and therefore their livelihoods, or hide their condition to protect their jobs [6,7].

The most common signs of mental health risks that pilots tend to exhibit are uncharacteristic behaviors, a change in personality, expression of anger, anxiety, moodiness, or acting like a different individual [8]. In addition, they may experience social withdrawal, isolation, a lack of self-care, panic, the feeling of being overwhelmed, and a sense of hopelessness [9,10]. In aviation, mental health issues may have consequences such as discrimination and stigma, grounding, loss of income, and fear of employment loss. Other outcomes associated with mental health issues are self-confidence and self-esteem problems, reluctance to seek help, failure to disclose the condition, increased stress, and isolation. All of these increase aviation safety risks as they may lead to extreme cases.

The current strategy, from a regulatory point of view, can be to err on the side of caution by removing a pilot from flight status [11]. Research analyzing literature sources has shown that the magnitude of mental health problems in aviation is mostly unknown because of the reluctance to report issues [9]. However, mental health may be affected by a variety of personal and occupational factors. Most mental disorders among pilots relate to anxiety, depression, and substance dependence [9]. Thus, it is critical to make sure that pilots have resources and routines to prevent mental health issues and get timely treatment for such conditions to help reduce human error, which is a key safety factor.

The causes of anxiety, depression, and other conditions that affect mental health are not well delineated. Research indicates that these are usually caused by a combination of environmental, genetic, biological, physical, and psychological factors. These factors include stress, trauma, personal or family history of depression, certain mental illnesses, anxiety, significant life changes, the use of medication, or poor relationships with colleagues [3].

Pilots have a unique working environment which significantly contributes to the risk of developing mental health conditions. The physical environment in which pilots operate is associated with an inordinate demand on mental and physical faculties [3]. Substantial restrictions in the workplace and limited movement and communication may lead to the development of mental health issues. Pilots spend long periods in isolation, which deprives them of the opportunity to regularly talk to their friends or family members [8]. Moreover, irregular work hours distort their sleep patterns, contributing to the risk of anxiety or depression which directly relates to fatigue and irregular sleep patterns. Additionally, lifestyle conditions, personal worries, and poor communication with colleagues may contribute to mental health challenges among pilots [8].

As demonstrated in the literature, pilots' mental health is critical for safe operations. Moreover, the literature has also shown that there are numerous barriers to pilots reporting potential mental health issues. The purpose of our research was to examine pilots' perceptions of mental health issues, the resources available to them, and the reasons they may or may not report issues. To better understand pilots' hesitation to discuss and report possible mental issues, the following research questions were formulated:

RQ1: What are pilots' perceptions of mental health issues? RQ2: What are pilots' perceptions of resources available to them? RQ3: What are the reasons pilots may not report issues?

## Materials and methods

For this study, the researchers used a qualitative, phenomenological approach to explore pilots' perceptions of mental health issues, resources available to them, and reasons they may or may not report issues. This methodology was deemed the best approach for this study because qualitative methodology generates an in-depth understanding of human experience with complex and fluid contexts [12].

Sample and procedures

A purposeful sample of 21 pilots, from five different Part 121 major airlines in the United States, were interviewed to better understand their perceptions of mental health issues, resources available to them, and reasons they may or may not report issues. Purposeful sampling was regarded as most appropriate as it allows better matching of the sample to the objectives of the research. A better matching of the sample creates better trustworthiness of the data and results [13].

Only Part 121 major airline pilots were interviewed for this research. We obtained approval from Embry-Riddle Aeronautical University IRB (approval #24-071) before any solicitation of participants or data collection. Initial requests were made through social media via the individual airline’s pilot forums. From the forums, participants volunteered to be interviewed. To ensure the participants had an adequate knowledge of mental health issues to be included in the research, each participant was required to be an active pilot and employed with their respective airline for at least the past 4 years. Moreover, each participant must have stated they had knowledge of their airline’s mental health services and a willingness to share their experiences. Any pilot who did not meet these criteria was excluded from our study.

The purposeful sample ensured a cross-section of pilots from different age groups, backgrounds, and airlines were included. Participants were selected to ensure the representation of both domestic and international pilots with a range of experience at five different airlines. The purposeful sampling and inclusion of both domestic and international pilots helped ensure the results set a foundation for generalizing to the larger population. E-mails were sent to the participants explaining the intent of the interview and requesting their participation. Once a participant agreed to be interviewed, IRB-approved consent letters were e-mailed to each participant, and signed consent letters were returned before the interview.

The semi-structured interview questions for this study were designed by the researchers based on Silva et al. (2022) [3] and the FAA’s 2023 [6] descriptions of how to address mental health challenges and increase opportunities to improve safety. Specifically, our survey addressed common areas of mental health including trust, fear, stigma, and the reporting venues available in mental health reporting. The questions were field-tested with three senior pilots (all were captains and had experience in the field of human factors teaching/training); minor wording changes were made to the final interview questions.

Interviews were conducted online via Teams or Skype. Interviews lasted between 31 and 47 minutes, with an average of 41 minutes. The interviews were conducted and recorded during the first two weeks of December 2023. After each interview, transcripts were provided to each participant to ensure accuracy. Once all transcripts were verified to be accurate, the transcripts were analyzed using NVivo 14 software. NVivo software is used to organize, analyze, code, and reveal themes that emerge in qualitative data [14]. Participant demographics are detailed in the Results section. Interview questions are provided in the Appendix.

## Results

Demographics

The purpose of this research was to better understand Part 121 pilots' perceptions of mental health issues, resources available to them, and reasons they may or may not report issues. After each interview, transcripts were provided to each participant to ensure accuracy. The transcripts were analyzed using NVivo 14 software. NVivo software is used to organize, analyze, code, and reveal themes that emerge in qualitative data [14]. Twenty-one pilots participated in the interviews during December of 2023. Of them, nine (42.9%) were previously military only, four (19.0%) had previous military and commuter airline experience, seven (33.3%) were previously commuter only, and one (4.8%) was previously corporate with neither military nor commuter flying.

Regarding age, three (14.3%) were in their 20s, five (23.8%) were in their 30s, five (23.8%) were in their 40s, six (28.6%) were in their 50s, and two (9.5%) were in their 60s. In addition, five (24%) identified as female and 16 (76%) identified as male. Moreover, 11 (52%) had flown for a commuter airline and 10 (48%) had not previously flown for a commuter airline. The purposeful sampling method ensured that various age groups and previous backgrounds were represented (Table 1).

**Table 1:** Demographics of the participants

Participant	Age, years	Years at 121 carrier	Previous military pilot?	Previous commuter pilot?	Airline
1	62	26	Yes	Yes	B
2	38	16	No	Yes	B
3	45	3	Yes	No	E
4	29	6	No	Yes	A
5	36	4	Yes	No	A
6	57	24	Yes	No	B
7	28	7	No	Yes	C
8	49	7	Yes	No	D
9	51	20	No	Yes	A
10	63	31	Yes	No	B
11	54	11	Yes	No	C
12	43	19	Yes	Yes	A
13	34	9	No	No	C
14	40	16	Yes	No	D
15	56	28	No	Yes	B
16	37	11	Yes	No	A
17	49	18	No	Yes	D
18	55	27	Yes	Yes	E
19	52	8	Yes	No	D
20	39	15	No	Yes	B
21	28	7	Yes	Yes	C

RQ1: What are pilots' perceptions of mental health issues?

All pilots indicated they had knowledge of mental health issues among pilots in the industry; however, few had specific knowledge of industry-wide issues, and that knowledge was usually limited to news items or those obtained word-of-mouth from other pilots. The most commonly mentioned mental health issues (that they were aware of) were depression (mentioned by 100% of the participants), family problems (mentioned by 90%), fatigue (mentioned by 67%), and financial (mentioned by 43%).

Theme 1: Pilots Avoid Discussing Mental Health Issues for Fear of Repercussions

For example, P15 stated, “I know they exist but no one would talk about it.” P5 reiterated “Be careful what you say to anyone-it can only hurt you…you say the wrong thing and you are in trouble.” P1 highlighted “The FAA doc is just like the flight surgeon-the best you will do is keep your medical but they can quickly take it away.”

Three participants highlighted their reluctance to discuss issues, as P7 stated, “Rarely talk about it. I know they exist but you are just kinda expected to deal with it.” P7 and P20 had similar responses, “Especially at the commuters you NEVER discussed it because you don’t want to highlight anything that might keep you from getting a job at the majors…managers at the commuters will do anything to keep you from leaving so you never talk to anyone about possible issues-they will try and use that against you to keep you there and prevent you from going to a major airline.”

The majority (71%) figured they were on their own. P13 stated, “You just have to figure out how to manage sleep and stuff. The funny thing is that everything happens when you are away from home…like the AC going out or the car not starting…causes more stress because you're gone from home”, while P11 said, “Being gone from home, poor sleep, don’t exercise like I should, worried that I should be home when I am not, all add to the stress of the job. Just deal with it.”

Nineteen of 21 (90%), including P6 and P9, shared concerns about even discussing the topic, “Same at the airline as in the military; do not highlight yourself. Maybe talk to a close friend, but be sure they won’t say anything. Just survive” and “I would be very suspicious of anyone who brought the subject up.”

RQ2: What are pilots' perceptions of resources available to them?

Overall, pilots had a positive impression of company resources available to them and were glad the resources existed, both within their respective airline companies and within their respective pilot groups, although 17 (81%) would not take advantage of the resources. Many had positive impressions that their aviation medical examiner (AME; a doctor who issues a pilot their medical certificate to allow them to fly) would do everything possible to help, but also realized the AMEs were bound by tighter regulations.

Theme 2: Although Resources Exist, Pilots Generally Distrust the Confidentiality of the Reporting Systems

P1 stated, “Officially, I know my company has resources, but I am afraid that if I use them, I will be highlighted" [to management, the FAA, and other pilots] while P4 expanded on this thought by saying, “I think my company does want to keep me flying but I worry that if I use resources, they are required to report most anything to the FAA and then I will lose my license while the FAA ‘tries to figure it out.’”

Participants stated that their AMEs were aware of the potential issues and tried to help whenever possible. P12 stated “My AME looked at my application and said, “Hmmm, check this answer before I complete your exam." whereas P17 highlighted, “My AME always personally checks the EKG to make sure “no yo-yo in the FAA office misinterprets anything and causes you a problem.”

Another example of distrust was offered by P3 stating “The idea of peer-to-peer is great, but I wonder about the qualification of 'just another pilot' talking to me would rather have a professional.” Many pilots felt that the pilots running peer-to-peer services were there either because they were zealots for counseling or still dealing with issues themselves. Although the pilots were grateful for the resources, 16 (76%) still distrusted the process as highlighted by P16 when they stated “I know there are probably some great resources available, but I am afraid my company or AME will find out and then I am screwed.”

P6 was the only pilot to have used company resources. “I talked with company EAP [employee assistance programs] about sleepless nights, due to depression, from my mother’s passing. They were great and gave me time off until things settled down. I am very grateful for their help but will always wonder if somewhere down the road some FAA person will find out about it and ground me for a re-evaluation.”

RQ3: What are the reasons pilots may not report issues?

Every pilot interviewed stated they believed that reporting any mental health issue could threaten their license, regardless of FAA or company statements to the contrary.

Theme 3: Pilots Honestly Believe That Reporting Any Mental Health Issue Will Be Devastating to Their Careers.

As P18 reported, “Seriously? I have heard horror stories about guys losing their license and costing them tens of thousands of dollars just to be permanently denied and grounded.” P6’s philosophy is, “Stay out of the system [do not highlight yourself with any possible medical issue]. Everything is fine. Report nothing because it will only hurt you. Yes, no one should fly if they are unfit, but to report something will cost you your job.” P7 reiterated, “No trust at all in the system to actually work with me.” Pilots reported that once something is reported, it would cost them money for additional testing, possible loss of income while waiting for test results, litigation fees, and quite possibly loss of their job.

Others echoed this sentiment. P8 highlighted, “Did not report my divorce (even though we saw a medical professional and should have reported it), even though it actually made us both happier, because I never knew what some FAA person might do.” P19 said if there is an issue “Become John Doe, pay out of pocket, get it fixed, and move on.” Finally, numerous participants stated that they “Report everything as 'no change' from my last medical, no matter what.”

## Discussion

Based on the results of the interviews, the topic of mental health is still avoided and feared by pilots. Although pilots understand that mental health is important, and should be discussed, few are willing to engage in any discussion for fear that they may be highlighted as “having a problem.” Moreover, there may be instances in which some AMEs are “leading” pilots to answer the “right” questions to keep them flying rather than assessing their condition accurately.

All pilots stated that either they had personal experience with mental health issues or personal knowledge of another pilot who had experienced a mental health issue. All pilots interviewed acknowledged that mental health issues are a concern but were hesitant to discuss mental health problems. This aligns with Newstex’s findings [7] that “His story speaks to a larger problem in the profession: Many pilots would rather ignore or hide their mental health problems than disclose their condition and risk their livelihood. As a result, pilots who may be safety risks remain in the air while those who have admitted they need help face a costly, time-consuming, and opaque process to meet the FAAs standards for medical fitness.” All participants felt it was still a taboo topic and that even mentioning it may highlight them in a negative way.

All pilots discussed their knowledge of the company and FAA resources available to them. Of note, 90% of the pilots felt they understood the resources available to them; however, most (81%) would not take advantage of these resources. Pilots generally did not trust the resources would be kept confidential and therefore would not use them. These feelings are similar to Weis’ findings [15]; one pilot stated, "You can't go see a therapist. You can't go see a counselor, you are constantly worried about not only losing the certificate in your pocket and the ability to feed your family, but I'll be very honest with you, you're going to lose your identity, and that fear is so strong that it just, it tears you apart." Having resources available, but being fearful of using them, simply adds to the frustration.

In addition, all pilots expressed hesitation to report mental health issues for fear of being grounded and possibly losing their jobs. Moreover, every pilot was reluctant to even discuss the topic for fear of possible investigations. All pilots hoped this situation would change soon. This aligns with with FAA Administrator Whitaker [9] who stated “Our focus is certainly going to be on safety in the cockpit, but I think we need to have a system that allows people to be more forthcoming and have treatment for issues that shouldn’t keep you out of the cockpit.” Recently, the FAA Aviation Rulemaking Committee released Task E [16], stating “Discuss and develop recommendations for mental health education programs….the FAA and the aviation industry could implement to improve awareness and recognition of mental health issues, reduce stigmas, and promote available resources to encourage voluntary self-disclosure in a confidential and protected environment, and assist with resolving mental health problems.” Creating this type of environment, where pilots feel they can seek treatment without losing their jobs, would be a great way forward to getting a better assessment of pilots' mental health.

Many obstacles discourage pilots from reporting their mental health issues at work. Long work hours make seeking treatment difficult, if not impractical [6]. Stigma is another aspect that prevents pilots from getting timely treatment as they are unwilling to report their mental health conditions. Other barriers to seeking professional and timely treatment are increased social withdrawal among individuals who experience anxiety, depression, and concerns toward treatment [6]. Mental health problems tend to increase in high-stress work situations. The financial consequences of employment interruption (losing the ability to fly) are another barrier that can prevent pilots from acknowledging mental health issues.

The coronavirus disease 2019 (COVID-19) pandemic has affected many industries, aviation in particular. The stress associated with the pandemic has affected all employees [17]. Isolation, ambivalence, and loss are salient and frequently discussed topics amidst concerns for risk and loss exposure. Empirical research indicates that aviation workers usually experience high levels of anxiety and depression, and they have to cope with a variety of mechanisms to deal with their mental issues. However, different barriers prevent them from reporting their mental health problems at work [8]. Unfortunately, research conducted by Cahill et al. [18,19] has shown that most pilots claim that their well-being, emotional well-being in particular, is not a priority for their organizations.

As Cahill et al. [20] stated, “Organizations are implementing a multi-strategy approach; however, the focus appears to be on secondary and tertiary interventions, and not primary interventions (i.e., preventative). Very few examples of primary interventions were provided.” Support is required not only for the current pilots but also for the ones who have lost their employment [18]. The working environment and working hours do not allow pilots to get proper treatment. Therefore, actively seeking assistance as a first step is an effective option.

The results of these interviews validate National Transportation Safety Board chair Jennifer Homendy’s statement: “The safety risk comes from a culture of silence around mental health” [2]. Some progress has been made, such as approving a handful of new drugs and treatments to allow pilots to continue to fly. Even though steps have been taken to encourage reporting and reduce barriers to retaining a medical certificate, pilots continue to steadfastly reiterate that nothing is discussed or disclosed through any official channel.

Limitations

This study was limited to pilots currently flying at US Part 121 operations. It did not include non-Part 121 pilot operations. The purposeful sample ensured the inclusion of a cross-section of pilots of varying ages, different backgrounds, and different airlines. The purposeful sampling and inclusion of both domestic and international pilots helped ensure that the results set a foundation for generalizing them to the larger population.

Recommendations

This research highlights several areas still preventing pilots from honestly discussing and disclosing mental health issues. Therefore, the following recommendations are put forth:

(1) Eliminate factors that prevent pilots from reporting mental health issues. If the health issues are not reported, these issues can continue to manifest with deadly consequences. This recommendation highlights the 2024 FAA Mental Health and Aviation Medical Clearances Aviation Rulemaking Committee report, Task A, acknowledging that a lack of trust, fear, and perceived stigma are reasons pilots do not report mental health issues [16].

(2) Pilots' potential health-related issues and company/pilot resources should be presented and discussed in yearly training. Confidentiality of the reporting sources (within legal limits) should be highlighted during training. Examples should include a wide range of experiences and conditions, as mental health stigma often paints a narrow picture of what mental health strain looks like. This is also in line with the 2024 FAA Mental Health and Aviation Medical Clearances Aviation Rulemaking Committee report emphasizing company culture as a venue to create a stronger mental health environment.

(3) Stronger pilot-to-pilot peer counseling resources (within the pilot groups and across airlines) need to be created to encourage early discussion of potential issues.

(4) Clear descriptions of pathways for mental issue resolutions need to be established. This recommendation is also in line with the 2024 FAA Mental Health and Aviation Medical Clearances Aviation Rulemaking Committee report to encourage pilots to seek treatment while still assuring safety. For example, a pilot needing time off for a divorce can expect a certain process, a pilot seeking help for depression can expect this path, etc. This could include a standard “cooling-off” period, such as removing the pilot from the cockpit, but for a pre-established period during the difficult mental condition, with a reevaluation and return to the cockpit at a specified time. Although there is no perfect path for every situation, pilots need to have realistic expectations of processes, time frames, and how their medical certificate may or may not be affected.

Future research

As this research encompassed a purposeful sample of Part 121 major airline pilots, the research should be expanded to include a greater sampling of pilots to gain deeper insights into the reasons why pilots are unwilling to disclose mental health issues. Moreover, since airlines include numerous employee groups, research should also include flight attendants, dispatchers, and mechanics as their skill sets are also critical to safe operations. Finally, this research should be expanded to include analysis by age, race, and gender demographics.

## Conclusions

Pilots are scared. They are scared that if they report, even mention, mental health issues, they instantly risk their jobs, so most choose to not disclose any issues. We investigated pilots' perceptions of mental health issues, resources available to them, and reasons they may not report issues. Regardless of their backgrounds both before and during their time with a Part 121 carrier, pilots had a general distrust of the current processes and therefore would neither discuss nor seek treatment for mental health issues. This aligns with previous research on pilots’ fearful attitudes toward reporting issues. Pilots consistently admitted an unwillingness to even discuss mental health issues lest they permanently lose their license. They focused on the stigma associated with reporting almost any mental health issue, regardless of the severity or specifics of potential conditions.

Recognizing the pivotal role of mental health in aviation safety, regulatory bodies are increasingly incorporating mental health considerations into their frameworks. The integration of mental health assessments, support mechanisms, and training programs into aviation regulations reflects a proactive approach to ensuring the mental well-being of aviation professionals. Pilots need support mechanisms that directly address mental health challenges, limit adverse career impacts, and when appropriate, return pilots to the cockpit. Fear of repercussions from reporting mental health issues may prevent some pilots from receiving help with conditions early in their development. Until the culture and stigmas against discussing mental health issues change, pilots will continue to hide issues that may affect safe airline operations.

## Appendices

Interview questionnaire

1. How do you prioritize physical and mental health maintenance, given the demands of your profession?

2. What do you think are some instances where personal matters could affect your mental state during a flight?

3. How do you mitigate the effects of sleep disruption and circadian rhythm disturbances?

4. How important is peer support in maintaining mental health within the aviation community?

5. Are there any specific aspects of your job that you find particularly challenging from a mental health perspective?

6. How do you prepare mentally for intense training sessions or simulator exercises?

7. How comfortable are you in your knowledge of what needs to be reported to your AME or company?

8. If you were to have a situation that affected your mental health, such as consistent lack of sleep, an ill family member, or a tense family situation at home:

a. Would you talk to a friend about it?

b. Does it need to be reported to your AME?

c. Does it need to be reported to your company?

9. What do you think would be the results of reporting to:

a. Your AME?

b. Your company?

10. What are your hesitations about reporting a condition to your AME or company?

11. What would help alleviate your concerns about reporting conditions to your AME or company?

12. How do you think training programs can be enhanced to better address mental health issues in the aviation industry?
